# Closed vs. Open-Skill Contexts in Basketball: Insights into Reactive and Nonreactive Short Distance Sprint Performance More Closely Aligned with Game Demands  

**DOI:** 10.3390/sports14030115

**Published:** 2026-03-13

**Authors:** Asaf Shalom, Roni Gottlieb, Julio Calleja-Gonzalez

**Affiliations:** 1 Department of Physical Education, The Research Center for Sports and Physical Activity, Tel Hai Academic College, Kiryat Shmona 1220800, Israel; 2 Wingate Institute, The Academic College Levinsky-Wingate, Wingate Campus, Netanya 4290200, Israel; ronigot23@gmail.com; 3 Department of Physical Education and Sports, Faculty of Education and Sport, University of the Basque Country, UPV/EHU, 01007 Vitoria-Gasteiz, Spain

**Keywords:** basketball, reactive, explosive power, sprint, sport-specific performance

## Abstract

Background: Basketball requires frequent short-distance sprints performed under both predictable (closed-skill) and unpredictable (open-skill) conditions. Objectives: This study compared sprint performance between closed- and open-skill conditions in 37 professional male basketball players aged 16–18 years. We aimed to determine whether sprint times differ between conditions and distances, test for a condition-by-distance interaction, and evaluate whether player rankings remain consistent across conditions. Methods: All players completed 5 m and 10 m sprints under both closed- and open-skill formats. Performance was analyzed using repeated-measures analysis of variance (ANOVA) for main effects and interactions, paired-samples *t*-tests for condition-specific comparisons, and correlation analyses to examine ranking consistency. Results: Sprint times were significantly slower in open-skill compared to closed-skill conditions at both distances (*p* < 0.001), indicating a clear performance decrement when responding to a visual stimulus. No significant condition-by-distance interaction was observed, despite a descriptively greater slowdown at shorter distances. Ranking consistency between conditions was low, indicating that faster closed-skill performers did not necessarily maintain their advantage in open-skill scenarios. Conclusions: These findings suggest that open-skill sprinting may reflect a distinct performance profile integrating physical acceleration and perceptual–cognitive processing. Including reactive sprint assessments in studies may enhance the sport-specific evaluation of explosive speed in basketball.

## 1. Introduction

Basketball is considered one of the most physiologically demanding sports, due to the fact that players are required to perform repeated high-intensity explosive actions [1]. Key movements in the game, such as rapid accelerations, sharp decelerations, quick changes in direction, and physical contact situations, rely mainly on the alactic anaerobic energy system, which is the dominant contributor of energy during short and intense efforts [1,2,3,4,5,6].

A central characteristic of this demand is the performance of short sprints, often repeated many times within only a few minutes of play, and success in these actions may determine the outcome of an offensive or defensive sequence [1,6,7,8]. These sprints do not occur in controlled laboratory conditions but rather in a dynamic and competitive environment, in which players must react quickly to external stimuli such as opponent movements, ball position, or coaching instructions [9,10].

These actions reflect open skills that require not only physiological capacity but also cognitive responses and quick decision-making under pressure [11,12,13].

In recent decades, sports science research has devoted considerable attention to examining the effects of various training methods and warm-up protocols on anaerobic performance [9,10,14]. One of the most extensively studied areas in this context is the assessment of explosive power through short sprint tests [2,15,16,17,18].

Most studies tend to evaluate these performances under relatively controlled conditions, defined as closed skills [1,2]. In such situations, testing is conducted using fixed-distance measurements, typically 5, 10, 15, or 20 m, depending on the demands of the sport or the specific research protocol [2,3,19].

This approach allows the isolation of the physiological performance factor, enabling the evaluation of the athlete’s “pure” ability to develop explosive horizontal force, without the influence of additional factors [1,2]. However, it is important to note that such tests do not fully represent the complexity of competitive demands, as real-game sprinting inevitably involves responding to external stimuli, dealing with opponent pressure, and making immediate decisions [9,10,13,20].

In line with this development, scientific recognition has increasingly acknowledged in recent years that physiological components alone cannot fully explain performance quality in team sports in general, and in basketball in particular [9]. Basketball players are required to react quickly to changing stimuli, process information in real time, and make tactical decisions while under continuous physical load [13].

The combination of physiological and cognitive demands reflects the complexity of the modern game and has led sports scientists to explore training and testing methods that integrate both aspects [9]. The emerging literature indicates, for example, that cognitive–motor dual-task (CMDT) activities may better simulate game conditions and help assess how players combine explosive power with information processing and decision-making abilities [9,13,20,21,22]. This approach is applied not only in training but also in the development of warm-up and preparation protocols aimed at sharpening players’ responsiveness to changing situations [14,23].

In light of the above, the main objective of the present study was to examine the specific influence of skill conditions on short, high-intensity sprint performance in basketball, which reflects the critical demands of professional games. For this purpose, we selected sprint distances of only 5 and 10 m, as they represent the most direct and powerful expression of horizontal explosive power and are particularly relevant to key game actions such as driving to the basket or executing rapid defensive coverage.

Specifically, this study aimed to (1) determine whether sprint time in an open-skill condition, in which the player must respond to an external stimulus, differs from sprint time in a closed-skill condition performed without reactive or unpredictable elements, and (2) determine the degree of consistency between the two conditions in order to evaluate whether strong performance in a closed-skill setting is maintained in an open-skill context or reflects a distinct ability requiring different physiological and cognitive capacities. In addition, the study sought to (3) determine whether differences between open- and closed-skill sprint performance vary according to sprint distance, and (4) assess whether an interaction exists between distance and skill condition, indicating that the magnitude of the performance gap may change as a function of sprint length.

## 2. Materials and Methods

### 2.1. Participants

This study enrolled 37 male basketball players aged 16–18 years, all of whom were members of a competitive youth basketball club in Israel (mean ± standard deviation [SD]; height 182.3 ± 5.3 cm; mean ± SD body mass 73.2 ± 6.3 kg). Each player had participated in structured basketball training and official competitions for at least five years prior to enrollment. During the competitive season, the players’ weekly routine typically included five basketball practices, two strength and conditioning sessions, and one league game. Data collection was conducted during the early phase of the annual training cycle, which included the preseason period and the initial weeks of the competitive season. Four main inclusion criteria were applied: (a) participants were required to have attended at least 85% of the scheduled preseason training sessions; (b) fully participated throughout the previous competitive season; (c) be free from musculoskeletal injuries, pain, or medication use; and (d) be medically cleared for full participation in sport. All testing procedures were performed on the club’s basketball court, a familiar environment that enhanced ecological validity. Participants wore their own basketball shoes and standard training apparel during all assessments. The participants and their parents provided their informed consent before the start of the study, in accordance with the Declaration of Helsinki [24], after approval by the local ethical committee of the Levinsky-Wingate Academic College (Reference number: 435, 20 February 2024).

### 2.2. Testing Protocol

To examine differences in explosive sprint performance between open-skill and closed-skill conditions, as well as to address the additional aims outlined in the Introduction, all participants performed a standardized 20 min warm-up [14,23,25,26]. This warm-up emphasized gradual preparation for short, high-intensity actions and included dynamic movements aligned with basketball-specific demands. Following the warm-up, each participant completed sprint tests over 5 m and 10 m under both conditions. Performance times for both distances were recorded simultaneously using an electronic timing system (Microgate, Bolzano, Italy), as detailed below. Each participant performed two planned trials in each condition, resulting in four sprint performances in total. Sprint times for 5 m and 10 m were obtained from the same sprint trial. The best performance time within each condition for each distance was retained for statistical analysis. Given that sprinting represents a natural, game-related movement pattern, two trials were considered sufficient to capture maximal acceleration performance. The testing sequence was randomized, with some players starting with the open-skill condition and others starting with the closed-skill condition, thereby minimizing potential effects of learning, order-related bias, or fatigue and supporting the internal validity of the findings. A repeated trial was performed only if a trial was deemed invalid due to a clearly identified technical or execution error.

### 2.3. Assessment Procedures

#### 2.3.1. Sprint Testing Overview

All participants completed 5 m and 10 m sprint tests to assess short-distance acceleration and explosive speed. The two testing variations, closed-skill and open-skill, were performed on the same day. A 5 min passive recovery period was provided between all maximal sprint trials to support full recovery. Each sprint began from a standardized standing start position, with feet shoulder-width apart and the lead foot positioned 50 cm behind the starting line, in accordance with established methodological standards in sports science [27]. Researchers verified the starting posture before each attempt to maintain methodological consistency.

#### 2.3.2. Closed-Skill Sprint Test (CST)

In the closed-skill condition, participants performed maximal sprints without any reactive or cognitive component. They initiated movement voluntarily at their chosen moment. Two valid trials were completed by each participant, and the fastest time was retained for analysis. Times for both 5 m and 10 m were recorded simultaneously using synchronized Witty-GATE photocells. Six photocells were positioned in parallel rows to enhance timing precision and reduce measurement noise, as previously described [1,2,14].

#### 2.3.3. Open-Skill Sprint Test (OST)

**Figure 1:**
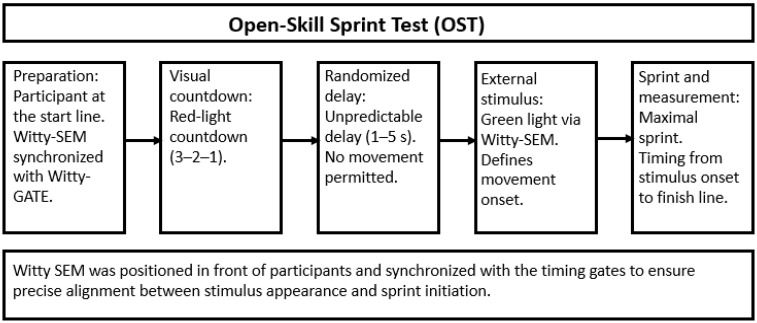
Flowchart of the OST protocol conducted in this study. OST = open-skill sprint test; s = seconds.

In the open-skill condition, an external visual stimulus determined the initiation of movement. A Witty SEM device displayed a red-light countdown (3–2–1), followed by a randomized delay of 1–5 s, following which the green light appeared. Participants were instructed to react immediately and sprint maximally once the green signal was presented. Timing started when the green light appeared. Therefore, the OST time includes the response to the visual stimulus, the reactive start, and the sprint over 5 m and 10 m. The same Witty GATE photocell configuration used in the CST was also applied in this condition to maintain consistency. The start timing gate was positioned on the baseline, whereas the lead foot was placed 50 cm behind a marked line located behind the baseline. A Witty SEM device positioned in front of participants served as the reactive cue, operating in synchronization with the timing gates to ensure precise alignment between stimulus appearance and sprint initiation [10,14]. False starts or anticipations were closely monitored by the research staff and coaches, and any invalid attempt was to be repeated; however, no false starts occurred in the study. Figure 1 presents a flowchart of the OST protocol. In addition, it should be clarified that in the OST, the open skill element was operationalized as a randomized psychomotor response to an external visual stimulus. Timing started at stimulus onset; therefore, the OST outcome reflects both response latency to the visual stimulus and sprint time to the finish line.

#### 2.3.4. Technology and Measurement Reliability

Sprint times over 5 m and 10 m were recorded using Witty GATE photocells (gate-to-gate time). In the open-skill condition, the Witty SEM device was synchronized with the timing system so that timing was triggered at stimulus onset, while the photocells captured the time to each distance. This configuration minimizes human error, enhances reliability, and ensures consistent timing procedures across both testing conditions. The synchronized operation of the systems in the open-skill condition allowed for valid and reliable assessment of reactive sprint performance [1,2,10,14,28]. Importantly, the system did not record reaction time as a separate component; rather, response and initiation were embedded within the OST outcome as part of the protocol.

### 2.4. Statistical Analysis

Descriptive statistics were calculated for all sprint variables and are reported as the mean ± SD and coefficient of variation (CV). Normality of all variables was evaluated using the Shapiro–Wilk test. To examine differences in sprint performance between conditions and distances, a two-way repeated-measures analysis of variance (ANOVA) was conducted with condition (closed-skill versus open-skill) and distance (5 m versus 10 m) as within-subject factors. ANOVA results are reported as F, degrees of freedom, *p* values, and partial eta squared (ηp^2^). When significant main effects were observed, planned paired-samples *t*-tests were used to compare closed- and open-skill performance separately for each distance. Paired test results are reported as t, degrees of freedom, *p* values, the mean within-subject difference with its SD, and 95% confidence intervals. When normality assumptions were not met, Wilcoxon matched-pairs tests were applied as non-parametric alternatives. Effect sizes for paired comparisons were expressed as Cohen’s dz, calculated as the mean value of within-subject differences divided by the SD of these differences. Effect sizes were interpreted as trivial (<0.20), small (0.20 to 0.49), moderate (0.50 to 0.79), large (0.80 to 1.19), or very large (≥1.20). As dz is standardized by the SD of within-subject differences, very large values may occur when the SD is small. Therefore, dz was interpreted alongside the mean within-subject difference and its 95% confidence interval. Pearson correlation coefficients were calculated to assess ranking consistency between closed- and open-skill conditions for each sprint distance, and their magnitudes were interpreted using conventional thresholds (for example, small, moderate, and large). Correlations are reported with 95% confidence intervals. An a priori power analysis using G*Power 3.1.9.7 (paired-samples *t*-test, large, expected effect size, α = 0.05, and power = 0.80) was used to estimate the minimum required sample size. This analysis indicated that a sample of approximately 20–25 players would be sufficient to detect large within-subject effects. The final sample of 37 players, therefore, provided more than adequate statistical power. Statistical significance was set at *p* < 0.05 (two-tailed). All statistical analyses were performed using JASP (version 0.19.3).

## 3. Results

### 3.1. Descriptive Results

Sprint performance for the 5 m and 10 m distances under both conditions is presented in Table 1. Across all players, sprint times were consistently faster in the closed-skill condition compared with the open-skill condition. Variability (represented by the CV) was low to moderate for all measures.

### 3.2. Differences Between Closed- and Open-Skill Conditions

A repeated-measures ANOVA showed a significant main effect of skill condition, with significantly slower sprint times in the open-skill condition, F(1,36) = 1383.78, *p* < 0.001, ηp^2^ = 0.975. A main effect of distance was also observed, F(1,36) = 10,707.80, *p* < 0.001, ηp^2^ = 0.997. As expected, sprint times were longer for 10 m than for 5 m across conditions. The distance by condition interaction was not significant, F(1,36) = 0.243, *p* = 0.625, ηp^2^ = 0.007. Planned comparisons confirmed this pattern for both distances:

- The 5 m distance: Open-skill, slower than closed-skill, mean difference = 0.759 s, SD of the differences = 0.121 s, 95% CI [0.719, 0.800] s, t(36) = 38.18, *p* < 0.001.
- The 10 m distance: Open-skill, slower than closed-skill, mean difference = 0.752 s, SD of the differences = 0.139 s, 95% CI [0.706, 0.799] s, t(36) = 32.86, *p* < 0.001.

**Figure 2:**
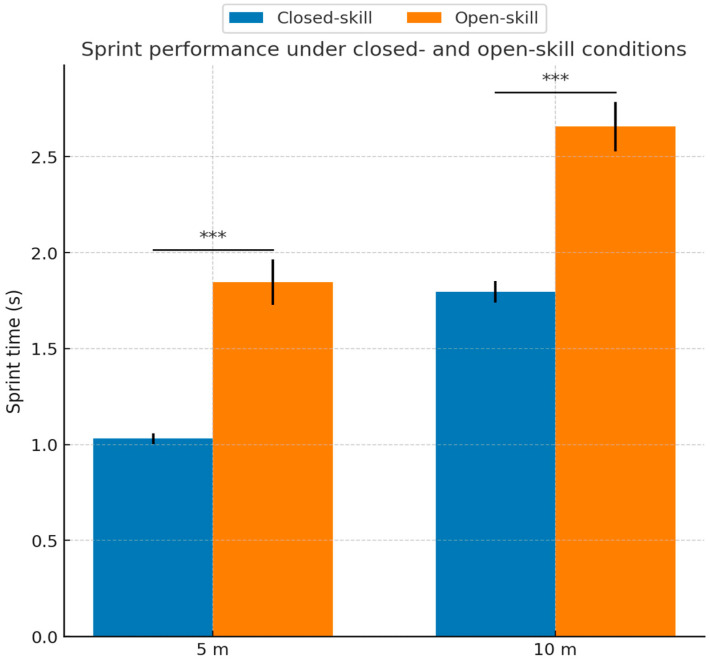
Mean values (seconds [s]) for sprint times for 5 m and 10 m under closed-skill and open-skill conditions (N = 37). Error bars represent standard deviation (SD); *** indicate a statistically significant difference between conditions, with a *p* < 0.001.

These findings suggest that adding an external, randomized visual stimulus is associated with slower short sprint performance. Accordingly, between-condition differences should be interpreted as reflecting the added randomized psychomotor response component embedded in OST timing, rather than sprint time alone. Figure 2 illustrates the mean differences in 5 m and 10 m sprint times between the two conditions.

### 3.3. Ranking Consistency Between Conditions

The comparison between closed- and open-skill 5 m sprint times showed a small and non-significant Pearson correlation (r = 0.110, 95% CI [−0.222, 0.419], *p* = 0.516). This indicates limited ranking consistency between conditions when a reactive visual stimulus is introduced. A similar pattern was found for the 10 m distance, with a small and non-significant Pearson correlation between closed- and open-skill times (r = 0.227, 95% CI [−0.105, 0.513], *p* = 0.177). This suggests limited ranking consistency between conditions when an external, unpredictable visual stimulus is introduced. Sprint times were longer for 10 m than for 5 m across conditions (*p* < 0.001).

## 4. Discussion

The present study examined differences in short-distance sprint performance between open-skill and closed-skill conditions in youth basketball players and also assessed whether players who perform well in a closed-skill setting maintain their advantage when they respond to an external stimulus. Although previous research has examined open- and closed-skill performance in sport in general and in basketball in particular [22,29,30], the current study offers, to the best of our knowledge, a relatively novel and in-depth examination of this question. The study builds on prior research that has highlighted the importance of perceptual–cognitive integration in sprint and agility performance and includes an explicit evaluation of whether players who excel in closed-skill conditions maintain their relative advantage when moving to a reactive, externally driven situation [15,31]. In addition, this study evaluates whether performance differences between conditions vary according to sprint distance and whether a distance × condition interaction could be identified.

Our findings demonstrate that sprinting in an open-skill context leads to a clear and meaningful decline in performance compared to a closed-skill context, both over 5 m and 10 m distances. This decline was statistically significant for both distances (*p* < 0.001), with very large effect sizes, indicating that the impact of adding a cognitive–perceptual demand is both robust and consistent across players. Although the descriptive slowdown appeared larger at 5 m, the interaction between distance and condition did not reach statistical significance in this sample. This suggests that the added cognitive–reactive demand impairs sprint performance similarly across short acceleration phases, and that the performance gap between open and closed conditions does not systematically widen as distance increases. As expected, 10 m sprint times were slower than 5 m sprint times across all players, reflecting the natural progression of acceleration mechanics. As described in the Methods and Results sections, timing in the open-skill condition started at stimulus onset; therefore, open-skill sprint times include a psychomotor response component in addition to sprinting time.

A central finding of this study is the relatively low between-player consistency across the two conditions. Players who were among the fastest under closed-skill conditions did not necessarily retain this advantage when required to respond to a visual stimulus. This suggests that open-skill sprinting recruits additional performance determinants beyond pure acceleration capacity. Determinants may include information processing speed, attentional control, decision-making, and subtle adjustments in starting mechanics driven by uncertainty regarding stimulus timing. The small and non-significant Pearson correlation values observed between closed- and open-skill sprint times support this interpretation, indicating that the ability to accelerate effectively in isolation does not fully predict performance under reactive constraints. Such cognitive–motor components have the potential to reorder performance rankings, meaning that “fastest at baseline” does not always translate into “fastest under pressure.”

These insights hold particular importance in basketball, a sport characterized by rapid perceptual demands, frequent changes in movement direction, and the need for instantaneous responses to unpredictable stimuli. In such environments, performance is not determined solely by physical output but by the integration between neuromuscular explosiveness and cognitive readiness. The current results highlight that evaluating only closed-skill sprint performance may overlook critical components that are essential for real-game execution. It is important to note that in this study, the open-skill sprint was defined as a response to an external, unpredictable visual stimulus. This mainly reflects reactive initiation, reaction time, and early acceleration, rather than full game-based perceptual demands.

Despite the relevance of this concept to game-based sports, it is surprising that much of the existing literature still focuses predominantly on closed-skill assessments. Traditional testing practices often fail to incorporate cognitive or reactive elements that are inherent to competitive performance [1,2,31]. However, recent studies have begun to shift this perspective, emphasizing the central role of cognition in modern basketball performance. Emerging evidence underscores the importance of integrating perceptual–cognitive demands into training approaches and identifying optimal warm-up strategies that prepare players both physically and cognitively for explosive actions [9,10,13,14,32,33]. The present findings reinforce the need for this shift by demonstrating that even short-distance accelerations are meaningfully altered when players must process and react to external information, highlighting the practical relevance of reactive-speed profiling in basketball.

Some studies have explored how heightened cognitive load affects perceived exertion and movement efficiency, suggesting that tasks requiring decision-making, inhibition control or rapid stimulus processing may increase the physiological and psychological demands placed on players [34]. This adds further support to the argument that open-skill performance represents a distinct and highly relevant domain of athletic ability that deserves greater emphasis in both evaluation and training [9,13,34].

In line with these models, the reduced ranking consistency observed in the present study suggests that reactive speed may not be explained by pure acceleration capacity alone, and likely reflects additional psychomotor demands associated with responding to an external, unpredictable stimulus. However, the specific cognitive mechanisms underlying this difference could not be inferred from the present data, as they were not directly assessed.

Overall, the findings of this study contribute to the growing body of evidence suggesting that sprint performance in open-skill contexts is not merely a derivative of closed-skill ability but may be condition-specific and reflects broader psychomotor demands. Basketball practitioners should consider incorporating reactive sprint assessments and reactive, externally cued drills into both testing batteries and training programs to more accurately reflect the demands of the sport.

### 4.1. Limitations

Several limitations should be acknowledged in interpreting the findings of this study. First, the sample consisted solely of 16- to 18-year-old male youth basketball players from a single competitive club, which limits the generalizability of the results to other age groups, competitive levels, or female players. In addition, biological maturation and playing position, which can influence sprint performance, were not assessed or controlled. Third, although the open-skill protocol introduced a reactive component via an external, unpredictable visual stimulus, it primarily captured reactive initiation, reaction time, and early acceleration. The protocol did not replicate the broader, multidimensional perceptual decision demands of basketball sprinting, such as contextual decision-making or opponent-related stimuli. Fourth, only linear sprint performance was examined. Important aspects of basketball-specific explosiveness, including reactive change-of-direction and vertical jumping in open-skill contexts, were not evaluated in this study. Fifth, using the best performance, a common approach in sprint testing to represent maximal performance, may slightly inflate effect estimates and does not fully capture within-player variability, despite the fact that each participant completed two maximal trials per condition with standardized recovery, resulting in four sprints in total. Finally, although the randomized order reduced learning and fatigue effects, the relatively small sample size may have limited the statistical power to detect subtle interaction effects, such as distance-dependent differences between conditions.

### 4.2. Future Research Directions

Future research should expand the examination of open- and closed-skill performance across broader populations, including female players, different playing positions, and multiple developmental stages. Incorporating reactive change-of-direction tasks and open-skill vertical jump assessments may provide a more comprehensive understanding of the psychomotor components that shape explosive performance in basketball. It may also be valuable to compare these patterns across different team sports to explore whether the magnitude and nature of open-skill performance decrements are sport-specific or represent generalizable characteristics of dynamic game environments. Additionally, future investigations should explore how cognitive load, attentional demands, and the perception of cognitive effort influence both physical performance and subjective responses, and whether targeted cognitive–motor training interventions can improve reactive explosiveness in competition-like scenarios.

## 5. Conclusions

A deeper examination of the findings suggests that the distinction between sprint performance in open- and closed-skill conditions reflects more than a simple difference in testing format. It may point to two potentially different performance profiles, one relying primarily on neuromuscular explosive power and another that may integrate perceptual and cognitive demands that are highly relevant to game-like situations in basketball. This understanding cautiously reinforces the importance of developing training methods that not only enhance pure acceleration capacity but also may cultivate cognitive abilities such as rapid information processing, selective attention, and effective decision-making under uncertainty, pending further evidence supporting this interpretation.

Although closed-skill assessments remain valuable for identifying baseline explosive potential, they do not provide a complete representation of an athlete’s functional ability in dynamic sport environments. Therefore, performance evaluation and training protocols should strive to incorporate a broader range of tasks that include open-skill elements, such as reactive sprinting, change-of-direction movements, and vertically oriented explosive actions triggered by external cues. Expanding assessments to compare performance across age groups, playing positions, and genders may further enrich our understanding of these capabilities.

Considering the broader landscape of game-based sports, it may also be beneficial for future research to examine whether similar patterns of performance differences emerge across other sport disciplines. Such comparison could contribute to a more comprehensive understanding of the psychomotor demands that distinguish open-skill and closed-skill performance and help refine sport-specific training and evaluation strategies. A longitudinal examination of how athletes adapt to reactive-speed training could help clarify how these abilities develop over time and how they can be best improved throughout the athlete development process.

## Figures and Tables

**Table 1:** Descriptive measurements for study participants (N = 37).

Distance (m)	N	Condition	Mean (s)	SD	CV (%)	Cohen’s dz	ES Magnitude
5 m	37	Closed-skill condition	1.02	0.03	3.2	—	
5 m	37	Open-skill condition	1.77	0.12	6.8	—	
5 m	37	Closed- versus open-skill comparison	—	—	—	6.28	Very large
10 m	37	Closed-skill condition	1.81	0.07	4	—	
10 m	37	Open-skill condition	2.56	0.14	5.3	—	
10 m	37	Closed- versus open-skill comparison	—	—	—	5.4	Very large

CV = coefficient of variation; ES = effect size; s = seconds; SD = standard deviation. Note: Cohen’s dz was calculated for paired comparisons as the mean of within-subject differences divided by the SD of these differences. Very large values occurred when the SD of the difference scores was small. Therefore, dz was interpreted alongside the mean within-subject difference and its 95% confidence interval, as reported in the following subsection of this section.

## Data Availability

The data presented in this study are available on request from the corresponding author due to ethical and privacy restrictions.

## Abbreviations

The following abbreviations are used in this manuscript:

CMDT	Cognitive motor dual task
CST	Closed-skill sprint test
OST	Open-skill sprint test
